# Biomarkers of Age-Related Frailty and Frailty Related to Diseases: An Exploratory, Cross-Sectional Analysis from the MAPT Study

**DOI:** 10.1007/s12603-022-1793-9

**Published:** 2022-05-04

**Authors:** D. Angioni, W.H. Lu, S. Sourdet, T. Macaron, C. Takeda, S. Guyonnet, J.F. Mangin, Y. Rolland, P. de Souto Barreto, B. Vellas, MAPT/DSA Group

**Affiliations:** 1 Gérontopôle of Toulouse, Toulouse University Hospital (CHU Toulouse), Toulouse, France; 2 Inserm UMR1295, University of Toulouse III, Toulouse, France; 3 Université Paris-Saclay, CEA, CNRS, Neurospin, UMR 9027, Gif-sur-Yvette, France; 4 CATI Multicenter neuroimaging platform, US 52, UAR 2031, CEA, CNRS, INSERM, APHP, ICM, Sorbonne Université, Paris, France

**Keywords:** Frailty related to diseases, age-related frailty, biomarkers, geroscience

## Abstract

**Background:**

Frailty may in most cases result from two main causes: the aging process (age-related frailty) and diseases (evolving chronic conditions or acute medical illnesses — disease-related frailty). The biological determinants characterizing these two main causes of frailty may be different.

**Objectives:**

The aim of this study is to compare the biological and neuroimaging profile of people without frailty, those with age-related frailty, and subjects with disease-related frailty in community-dwelling older adults.

**Material and Methods:**

We performed a secondary, cross-sectional analysis from the Multidomain Alzheimer Preventive Trial (MAPT). We included 1199 subjects without frailty throughout the 5-year follow-up, 82 subjects with incident age-related frailty, and 53 with incident disease-related frailty. Available blood biomarkers involved nutritional (eg, vitamin D, omega-3 fatty acids), inflammatory-related (IL-6, TNFR1, GDF15), neurodegenerative (eg, beta-amyloid, neurofilament light chain) and neuroimaging markers (MRI, Amyloid-PET).

**Results:**

Although not statistically significant, the results of the unadjusted model showed increasing gradients for inflammatory markers (GDF15, TNFR1) and decreasing gradients for nutritional and neuroimaging markers (omega 3 index, hippocampal volume) from age-related frailty participants to individuals with disease-related frailty. Considering the linear models we observed higher GDF15 values in disease-related frailty group compared to age-related frailty individuals [β = 242.8 (49.5, 436.2)]. We did not find any significant difference between subjects without frailty and those with age-related frailty. Subjects with disease-related frailty compared to subjects without frailty had lower values of DHA [β = −2.42 (−4.76, −0.08)], Omega 3 Index [β = −0.50 (−0.95, −0.06)] and hippocampal volume [β = −0.22 (−0.42,−0.02)]. They also had higher values of GDF15 [β = 246.1 (88.9, 403.4)] and TNFR1 [β = 157.5 (7.8, 307.2)].

**Conclusion:**

Age-related frailty and disease-related frailty may represent different degrees of frailty severity on a biological level. Further research is needed to identify biomarkers potentially able to distinguish these classifications of frailty.

## Abbreviation

Aβ: amyloid-beta
ApoE: Apolipoprotein E
CRP: C-reactive protein
DHA: docosahexaenoic acid
EPA: eicosapentaenoic acid
GDF15: growth differentiation factor 15
IL6: interleukin 6
MAPT: Multidomain Alzheimer Preventive Trial
MCP1: monocyte chemoattractant protein 1
SUVR: standard uptake value ratio
TNFR1: tumor necrosis factor receptor type 1
WF: without frailty
WMH: white matter hyperintensities

## Introduction

**A**lthough chronic diseases (1, 2) and acute medical events (3, 4) have been associated with physical frailty in cross-sectional and longitudinal studies, it is common to meet in clinical practice older adults becoming frail in the absence of significant medical events (5). For this reason, we have previously hypothesized that frailty in older adulthood could result from two main causes, the biological aging process itself (ie, age-related frailty) and evolving chronic conditions or acute illness (6) (ie, disease-related frailty).

Given the clinical relevance of frailty, there is an increasing interest to identify the markers for physical frailty (7). In the recent years, several markers such as nutritional (8), inflammatory (9), and neuroimaging markers (10) have been associated with incident frailty. However, as far as we know, no investigation examined differences in such biomarkers in people who became frail without overt disease (individuals rated as age-related frailty) and those with disease-related frailty. Since chronic conditions are often associated to a pro-inflammatory profile (11), nutritional deficiencies (12), and neurodegenerative markers (13), it is plausible to think that frail older adults with evolving diseases would cumulate the deleterious biological changes related to both aging and disease. If different biological profiles according to the main cause of frailty exist, this may ultimately inform different strategies to prevent/delay or reverse frailty.

The aim of this exploratory study is to compare the biological and neuro-imaging profile of people without frailty, those with age-related frailty, and subjects with disease-related frailty in community-dwelling older adults, from the Multidomain Alzheimer Preventive Trial.

## Methods

### Study population

The present study used data from the Multidomain Alzheimer Preventive Trial (MAPT) (14, 15). MAPT is a randomized controlled trial (registration: NCT00672685) aiming to assess the effects of multidomain interventions (nutritional and physical activity counselling, and cognitive training), omega-3 supplementation, or their combination on cognitive function over 3 years. The trial found no effect of these interventions compared to placebo on a composite cognitive score (16). MAPT participants were additionally followed for 2 observational years (no intervention). MAPT participants were community-dwelling individuals aged ≥ 70 years and meeting at least one of the following criteria: limitation in executing ≥1 Instrumental Activity of Daily Living (eg, cooking, shopping, using the phone, housekeeping), spontaneous memory complaints, slow gait speed (≤ 0.8 m/s). MAPT methods and procedures have been described elsewhere (14, 15, 16). MAPT respected the Declaration of Helsinki and was approved by the ethics committee (CPP SOOM II) in Toulouse. After signing informed consent, participants underwent clinical assessments, including frailty, at baseline and at 6, 12, 24, 36, 48 and 60 months.

### Frailty assessment

Physical Frailty status was assessed using Fried criteria (17): 1. Unintentional weight loss (more than 4.5 kg) in the past 12 months; 2. Fatigue measured by two questions from the Center for Epidemiologic Studies Depression Scale (CES-D); 3. Low handgrip strength based on the best of 3 measurements with preferred hand; 4. Slow walking speed based on the best of 2 measurements over 4 meters; 5. Low level of physical activity expressed in weekly energy expenditure considering time spent doing physical activities. Patients meeting 3 or more criteria were considered frail, those meeting 1 or 2 criteria pre-frail and those without any criterion robust. For the present study we considered:

• Subjects without frailty (WF): defined as robust or prefrail participants at baseline that did not become frail during the follow-up period;
• Subjects with incident frailty: robust or prefrail participants at baseline who became frail during the follow-up period. These subjects have been previously classified in age-related frailty, disease-related frailty, and frailty of uncertain origin. Given that the objective of the present work was to study the markers of age-related and disease-related frailty, we did not consider the subjects with frailty of uncertain origin in the current analysis. Classification methods and procedures have been described in our previous study (6). Summarily, clinical files were reviewed by two different clinicians using a standardized assessment method. Inconsistencies among the two raters and the cases of uncertain frailty were reconsidered by two more experienced raters in order to obtain the definitive classification.

### Blood biomarkers

A full description of measurement procedures is presented in the supplementary materials. Baseline omega-3 PUFAs were assessed in erythrocyte membranes, by measuring docosahexaenoic acid (DHA) and eicosapentaenoic acid (EPA). The omega-3 index was calculated as the sum of %DHA and %EPA (18). APOE ε4 carriers were defined by ApoE genotyping as having at least one ε4 allele.

All the other blood biomarkers were obtained from plasma samples. 25-hydroxyvitamin D (ng/mL) and homocysteine (µmol/L) were measured at baseline using a commercially available electro-chemiluminescence competitive binding assay (Cobas; Roche). Vitamin D status was classified into 3 groups (deficiency: <20 ng/mL; insufficiency: 20–29.9 ng/mL; sufficiency: ɥ30 ng/mL) according to previous research (19). Hyperhomocysteinemia was defined as homocysteine concentrations of more than 15 µmol/L (20). CRP levels (mg/L) were measured at baseline, 6 and 12 months by immunoturbidity according to standard protocols. Low-grade inflammation, according to previous research (21), was defined as having at least two CRP values within 3 to 10 mg/L in consecutive visits between baseline, 6- and 12-month visits. Acute inflammation was defined as having at least one CRP value >10 mg/L between baseline and the 12-month visit. At the 12-month, Aβ 42 and Aβ 40 levels were assayed by immunoprecipitation mass spectrometry. Progranulin (ng/mL) was measured at the 12-month visit by a Human Progranulin Quantikine ELISA kit (R&D Systems, DPGRN0) following the manufacturer's instructions. Neurofilament light chain (NfL) were assayed at the 12-month visit using the R-PLEX human neurofilament L antibody set (Meso Scale Discovery, F217X) at the 12-month visit. Growth differentiation factor 15 (GDF15) (pg/ml), Tumor Necrosis Receptor 1 (TNFR1) (pg/ml), Interleukin 6 (IL6) (pg/ml), and Monocyte chemoattractant protein 1 (MCP1) (pg/ml), were assayed using the fully automated immunoassay platform Ella (ProteinSimple/Biotechne, San Jose, CA, USA) at the 12-month visit. For biomarkers measured at 12 months, only participants who did not become frail, or who were not lost to follow-up before the 12-month visit, were considered for the current analysis.

### Neuroimaging markers

All participants recruited in the MAPT study were invited to join the Magnetic Resonance Imaging-MAPT ancillary study, that was conducted in 9 centers (Toulouse, Bordeaux, Dijon, Foix, Limoges, Lyon, Montpellier, Nice, and Tarbes), using a standardized protocol designed by the CATI, the French national platform for multicenter neuroimaging (22). In the present study, we considered MRI data of gray matter volume (cm3), hippocampal volume (cm^3^), the volume of White Matter Hyperintensity lesions (cm^3^) and global cortical thickness (mm). Global Cortical thickness was averaged from the two hemispheres. The hippocampal volume was calculated as the mean of the left and right hippocampal volume. For each measure, the imaging quality was scored and data with unreliable quality were excluded from our analysis. Only MRI data obtained before the date of incident frailty or the date of last frailty assessment were included.

All participants recruited in one of the 5 Positron Emission Tomography (PET) centers were invited to join the PET-MAPT ancillary study. [18F]-Florbetapir PET scans were performed for measuring brain amyloid-β load. In line with previous studies, significant brain amyloid-β deposits (ie, cortical SUVR positive) were defined as cortical SUVR ɥ 1.17 (23). Regional standard uptake value ratios (SUVRs) were obtained using semi-automated quantitative analysis with the whole cerebellum as the reference region. Cortical-to-cerebellar SUVRs were generated from the mean signal of six regions (frontal, parietal, temporal, precuneus, anterior cingulate, and posterior cingulate cortical regions). In the current analysis, we only considered participants, whose PET results were obtained before the date of frailty onset or their last frailty assessment visit.

### Statistical analysis

Descriptive statistics were presented as median and IQR, or frequencies and percentages. Chi-square/Fisher exact test and Kruskal-Wallis test were used to compare baseline characteristics and biomarkers according to the frailty status (without frailty, age-related frailty, disease-related frailty). We applied multivariate linear regressions to examine associations between biological markers (as the dependent variables) and the frailty status adjusted for age, sex and MAPT groups. For MRI variables, linear mixed-effect models (with random effect on study center) were conducted with adjustment for age, sex, MAPT groups, and total intracranial volume. For all linear models, the distribution of the residuals was assessed by visual inspection and the value of biomarker was log-transformed if the residuals deviated from a normal distribution. Logistic regressions (adjusted for age, sex and MAPT groups) were performed to evaluate associations of the frailty status with biomarkers in categorical measures. Statistical significance was defined as p < 0.05; data were analyzed by using SAS, version 9.4 (SAS Institute, Inc., Cary, NC).

## Results

Among 1679 subjects enrolled in MAPT study, 91 and 143 participants were excluded due to missing data from baseline and post-baseline frailty assessment respectively. Fifty-one participants with frailty at baseline were excluded. Among the remaining 1394, 1199 (86%) subjects did not become frail during the 5-year follow-up period, 82 (6%) subjects were rated as incident age-related frailty, and 53 (4%) as incident disease-related frailty. Sixty individuals with incident frailty were excluded from our analysis because it was not possible to classify them according to the main cause of frailty (ie, either age- or disease-related). Finally, a total of 1334 participants was included in this study. Subjects without frailty were younger at baseline when compared with other groups. We did not find any significant difference regarding sex and MAPT group between the three frailty groups (Table 1).

**Table 1:** Comparison of biomarkers according to frailty status

**Median (IQR) or n (%)**	**N**	**Without frailty (n=1199)**	**N**	**Age-related frailty (n=82)**	**N**	**Disease-related frailty (n=53)**	**p-value**
Age (years)	1199	74.0 (71.0, 77.0)	82	77.5 (74.0, 81.0)	53	78 (75.0, 81.0)	**< 0.001**
Sex (male)	1199	436 (36.4 %)	82	25 (30.5 %)	53	15 (28.3 %)	0.292
MAPT group	1199		82		53		0.718
Multidomain intervention + Omega 3		302 (25.2%)		21 (25.6 %)		10 (18.9%)	
Omega 3		287 (24.0 %)		23 (28.1 %)		17 (32.1 %)	
Multidomain intervention		317 (26.4 %)		17 (20.7 %)		13 (24.5 %)	
Placebo		293 (24.4 %)		21 (25.6 %)		13 (24.5 %)	
**Biological markers**							
25-hydroxyvitamin D (ng/mL)	515	23 (15, 32)	37	22 (14, 26)	36	18 (12, 35)	0.281
Deficiency (< 20 ng/mL)		197 (38.3%)		15 (40.6%)		21 (58.3%)	0.109
Insufficiency (20–29.9 ng/mL)		165 (32.0%)		13 (35.1%)		5 (13.9%)	
Sufficiency (ɥ 30 ng/mL)		153 (29.7%)		9 (24.3%)		10 (27.8%)	
Homocysteine (µmol/L)	512	14.8 (12.0, 17.8)	38	16.2 (12.7, 20.7)	36	16.7 (12.6, 18.9)	0.150
Hyper-homocysteinemia (>15 µmol/L)		239 (46.7%)		21 (55.3%)		20 (55.6%)	0.373
Erythrocyte membrane fatty acid	1135		80		50		
DHA (µg/g)		25.5 (20.3, 31.2)		25.6 (20.6, 29.7)		23.0 (17.7, 28.2)	0.168
EPA (µg/g)		4.7 (3.6, 6.3)		4.4 (3.1, 6.4)		4.3 (3.3, 6.2)	0.306
Omega-3 index (%DHA+%EPA)		5.9 (4.9, 6.9)		5.5 (4.6, 6.6)		5.3 (4.4, 6.5)	**0.009**
CRP (mg/L)	971		64		37		**< 0.001**
Normal		771 (79.4%)		48 (75.0%)		22 (59.5%)	
Low-grade inflammation		104 (10.7%)		11 (17.2%)		9 (24.3%)	
Acute inflammation		96 (9.9%)		5 (7.8%)		6 (16.2%)	
ApoE ε4 carrier	981	234 (23.9%)	73	14 (19.2%)	38	7 (18.4%)	0.506
Aβ42/40 ratio	332	0.110 (0.100, 0.120)	22	0.110 (0.100, 0.120)	20	0.110 (0.100, 0.120)	0.529
Aβ42/40 positive (≤0.107)		113 (34.0%)		7 (31.8%)		8 (40.0%)	0.836
Neurofilament light chain (pg/mL)	329	73.0 (55.4, 91.8)	22	79.3 (68.3, 93.0)	20	74.8 (63.7, 135.9)	0.210
Progranulin (ng/mL)	329	44.5 (38.3, 50.0)	22	44.0 (40.2, 48.5)	20	50.1 (42.9, 61.1)	**0.047**
GDF15 (pg/mL)	857	985 (795, 1264)	53	1009 (810, 1304)	33	1422 (1069, 1612)	**< 0.001**
TNFR1 (pg/mL)	858	1125.5 (947, 1364)	53	1256 (922, 1456)	33	1469 (1055, 1754)	**0.002**
IL6 (pg/mL)	857	2.5 (1.8, 3.6)	53	2.6 (1.7, 4.1)	33	3.6 (2.3, 4.5)	0.111
MCP1 (pg/mL)	858	202.5 (170, 246)	53	224 (176, 293)	32	213 (180, 288)	0.228
**Imaging markers**							
Cortical SUVR	203	1.13 (1.05, 1.28)	15	1.10 (0.95, 1.20)	5	1.35 (1.12, 1.39)	0.146
% SUVR positive (ɥ 1.17)		80 (39.4%)		5 (33.3%)		3 (60.0%)	0.545
Gray matter volume (cm^3^)	392	638.4 (599.5, 685.3)	20	633.4 (598.0, 664.9)	15	604.0 (558.8, 656.9)	0.079
Hippocampal volume (cm^3^)	395	3.5 (3.3, 3.8)	20	3.3 (3.2, 3.6)	15	3.2 (2.9, 3.5)	**0.003**
WMH volume (cm3)	379	9.7 (6.5, 15.8)	17	13.2 (8.6, 19.6)	15	12.5 (11.2, 21.6)	**0.033**
Cortical thickness, whole brain (mm)	383	2.36 (2.28, 2.43)	20	2.34 (2.23, 2.44)	13	2.34 (2.31, 2.40)	0.735


P-value determined using Chi-square/Fisher exact test for categorical variables or using Kruskal-Wallis test for continuous variables; Bold p-values indicate statistically significant differences (p < 0.05); Abbreviation: Aβ, amyloid-beta; ApoE, Apolipoprotein E; BMI, body mass index; CRP, C-reactive protein; DHA, docosahexaenoic acid; EPA, eicosapentaenoic acid; GDF15, growth differentiation factor 15; IL6, interleukin 6; MAPT, Multidomain Alzheimer Preventive Trial; MCP1, monocyte chemoattractant protein 1; SUVR, standard uptake value ratio; TNFR1, tumor necrosis factor receptor type 1; WMH, white matter hyperintensities.

Comparison of biomarkers between the three groups (without frailty, age-related frailty, disease-related frailty) with bivariate analysis are detailed in the Table 1. In the disease-related frailty group, we observed lower values of omega-3 index and hippocampal volume compared to both individuals without frailty and age-related frailty participants. Low-grade inflammation and acute inflammation were more common among participants with disease-related frailty compared to the two other groups. Significantly higher values of Progranulin, GDF15 and TNFR1 were found in the group frailty related to diseases compared to individuals without frailty and age-related frailty participants.

The results of the adjusted models are presented in the Table 2 (for continuous variables) and Table 3 (for binary variables). Subjects with disease-related frailty had higher GDF15 values compared with age-related frailty individuals. We did not find any significant difference between participants with age-related frailty and subjects without frailty. Subjects with disease-related frailty presented lower values of DHA, omega-3 Index, and hippocampal volume and higher values of GDF15 and TNFR1 compared to subjects without frailty.

**Table 2:** Multivariate linear regression* evaluating associations between biomarkers (dependent variables) and frailty status

	**Age-related frailty vs. without frailty β (95% CI); p-value**	**Disease-related frailty vs. without frailty β (95% CI); p-value**	**Disease-related frailty vs. age-related frailty β (95% CI); p-value**
**Biological markers**			
25-hydroxyvitamin D (ng/mL)	−1.52 (−5.74, 2.69); 0.478	−1.44 (−5.76, 2.87); 0.512	0.08 (5.60, 5.77); 0.977
Homocysteine (µmol/L)	0.70 (−1.09, 2.49); 0.442	0.10 (−1.75, 1.96); 0.913	−0.60 (−3.03, 1.83); 0.629
DHA (*µ*g/g)	−0.35 (−2.23, 1.52); 0.713	**−2.42 (−4.76, −0.08); 0.043**	−2.07 (−4.95, 0.82); 0.160
EPA (*µ*g/g)	−0.20 (−0.76, 0.35); 0.470	−0.28 (−0.97, 0.41); 0.431	−0.07 (−0.93, 0.78); 0.865
Omega-3 index (%DHA+%EPA)	−0.31 (−0.67, 0.04); 0.085	**−0.50 (−0.95, −0.06); 0.026**	−0.19 (−0.74, 0.35); 0.493
Plasma Aβ42/40	0.002 (−0.004, 0.008); 0.573	−0.003 (−0.010, 0.003); 0.341	−0.005 (−0.014, 0.004); 0.262
Neurofilament light chain (pg/mL)^2^	0.02 (−0.06, 0.09); 0.693	0.03 (−0.05, 0.12); 0.423	0.02 (−0.09, 0.13); 0.740
Progranulin (ng/mL)	−0.62 (−5.91, 4.67); 0.818	5.43 (−0.27, 11.14); 0.062	6.05 (−1.44, 13.54); 0.113
GDF15 (pg/mL)	3.3 (−120.5, 127.1); 0.958	**246.1 (88.9, 403.4); 0.002**	**242.8 (49.5, 436.2); 0.014**
TNFR1 (pg/mL)	14.9 (−102.9, 132.8); 0.804	**157.5 (7.8, 307.2); 0.039**	142.6 (−41.5, 326.7); 0.129
IL6 (pg/mL)^2^	−0.005 (−0.079, 0.070); 0.902	0.050 (−0.044, 0.144); 0.299	0.055 (−0.061, 0.171); 0.356
MCP1 (pg/mL)	15.5 (−8.3, 39.4); 0.201	−3.3 (−33.5, 27.0); 0.833	−18.8 (−56.0, 18.4); 0.322
**Imaging markers**			
Cortical SUVR	−0.05 (−0.14, 0.04); 0.303	0.12 (−0.04, 0.28); 0.133	0.17 (−0.01, 0.35); 0.057
Gray matter volume (cm^3^)	−3.78 (−9.11, 1.54); 0.163	−0.86 (−7.06, 5.33); 0.784	2.92 (−4.91, 10.75); 0.464
Hippocampal volume (cm^3^)	−0.06 (−0.24, 0.12); 0.505	**−0.22 (−0.42, −0.02); 0.035**	−0.16 (−0.42, 0.10); 0.224
WMH volume (cm^3^)	−1.37 (−5.78, 3.04); 0.542	2.47 (−2.27, 7.22); 0.306	3.84 (−2.36, 10.05); 0.224
Cortical thickness, whole brain (mm)	−0.03 (−0.08, 0.02); 0.234	−0.01 (−0.08, 0.05); 0.686	0.02 (−0.06, 0.10); 0.650


*Models using biomarkers as dependent variables with adjustment for age, sex and Multidomain Alzheimer Preventive Trial (MAPT) group. For MRI variables (gray matter, hippocampus, WMH and cortical thickness), mixed-effect model (with random effect on study center) was applied with adjustment for age, sex, MAPT groups, and total intracranial volume; 2. Value of biomarker was log transformed; Bold lines indicate statistically significant differences at p<0.05; Abbreviation: Aβ, amyloid-beta; CI, confidence interval; DHA, docosahexaenoic acid; EPA, eicosapentaenoic acid; GDF15, growth differentiation factor 15; IL6, interleukin 6; MCP1, monocyte chemoattractant protein 1; SUVR, standard uptake value ratio; TNFR1, tumor necrosis factor receptor 1; WMH, white matter hyperintensities.

**Table 3:** Logistic regression* evaluating association between biomarkers (dependent variables) and frailty status

	**N**	**Age-related frailty vs. without frailty OR (95% CI); p-value**	**Disease-related frailty vs. without frailty OR (95% CI); p-value**	**Disease-related frailty vs. age-related frailty OR (95% CI); p-value**
25-hydroxyvitamin D deficiency (< 20 ng/mL)	588	1.15 (0.48, 2.78); 0.750	1.33 (0.59, 3.01); 0.491	1.16 (0.37, 3.58); 0.803
Hyper-homocysteinemia (≥ 15 µmol/L)	586	1.18 (0.58, 2.39); 0.653	1.13 (0.55, 2.32); 0.748	0.96 (0.37, 2.49); 0.928
Low-grade inflammation	1072	1.71 (0.85, 3.44); 0.135	**3.10 (1.35, 7.11); 0.008**	1.82 (0.66, 5.04); 0.251
ApoE ε4 carrier	1092	0.79 (0.43, 1.46); 0.456	0.78 (0.33, 1.81); 0.555	0.98 (0.36, 2.68); 0.966
Low plasma Aβ42/40 (≤ 0.107)	374	0.71 (0.28, 1.80); 0.472	1.05 (0.40, 2.72); 0.928	1.47 (0.41, 5.28); 0.555
SUVR positive (≥ 1.17)	223	0.92 (0.28, 3.02); 0.893	2.26 (0.33, 15.50); 0.407	2.45 (0.28, 21.12); 0.415


*Adjusted for age, sex and Multidomain Alzheimer Preventive Trial (MAPT) group; Bold lines indicate statistically significant differences at p<0.05; Abbreviation: Aβ, amyloid-beta; APOE, Apolipoprotein E; OR, odds ratio; SUVR, standard uptake value ratio.

## Discussion

This is the first study aimed to compare the biological characteristics of subjects with incident age-related frailty, incident disease-related frailty, and subjects who did not become frail during a follow-up period of 5 years. GDF15 was the only marker for which we found a significant difference between the subjects with age-related frailty and those with disease-related frailty. Subjects without frailty did not differ from individuals with age-related frailty from a biological point of view. Subjects with disease-related frailty had worse levels of several inflammatory, nutritional and neuroimaging markers compared to people without frailty.

The higher GDF15 values in the group disease-related frailty, compared with age-related frailty, could be due to the higher comorbidity burden in the former group. In physiological conditions, GDF15 is a peptide expressed in multiple tissues at low concentrations. Nevertheless, this protein is overexpressed in several pathological conditions, such as cancer, systemic inflammation, metabolic and cardiovascular diseases (24). The role of GDF15 during diseases is few defined. GDF15 may play a protective role in several tissues, following inflammation, by mitigating the extent of damage (25). Moreover, GDF15 leads to appetite suppression, being potentially responsible for weight loss (26). Weight loss is a hallmark of frailty especially prevalent in the context of several chronic and acute medical conditions (27). Indeed, in our previous paper, we showed that the criterion weight loss was more common in people with disease-related frailty, compared to those with age-related frailty (6). Further studies are needed to clarify if GDF15 can distinguish these classifications of frailty in other populations.

Although not statistically significant, our results showed increasing gradients for inflammatory markers (CRP, GDF15, TNFR1) and decreasing gradients for nutritional (omega 3 index) and neuroimaging (hippocampal volume) markers from age-related frailty participants to individuals with disease-related frailty. This could suggest that, rather than opposite concepts, age-related frailty and disease-related frailty could represent different stratifications of frailty severity. Participants who became frail due to diseases are probably frailer than age-related frail subjects. This could be due to the fact that, in addition to the progressive effect of the aging process, they are weakened by the deleterious pathophysiological process linked to chronic comorbidities and acute diseases. In the future, a better knowledge of the hallmarks of aging (28) (ie, cellular senescence, epigenetic alterations, telomere attrition, etc.) and their biomarkers could help understand the pathophysiological mechanisms of these different classifications of frailty (ie, age-related and disease-related). This could ultimately permit identifying different strategies of prevention and treatment.

As expected, subjects with frailty related to diseases presented lower values of omega 3 and higher levels of inflammatory biomarkers than subjects without frailty. Low levels of omega 3 have been associated with several pathophysiological mechanisms (such as atherosclerosis, hypercoagulability, bone and muscle degeneration) and chronic diseases potentially leading to frailty (29, 30, 31). Low-grade inflammation is a common denominator of cardiovascular, metabolic, neurologic and systemic diseases (32). Furthermore, low-grade inflammation represents one of the most promising biological pathways that have been studied in the context of frailty pathogenesis (33).

This study extends the knowledge of the biological profile of individuals characterized as having developed age-related frailty and those with frailty related to diseases. Nevertheless, some limitations should be acknowledged. Firstly, this work is a secondary analysis of the MAPT study that was not specifically designed to measure the biomarkers of frailty. Frailty subtypes (ie, age-related or disease-related) classification was performed from clinical files in a retrospective manner. Furthermore, we investigated only biomarkers available in MAPT database. A non-biased approach is therefore needed to provide a comprehensive view of biological differences according to the main cause leading to frailty. We planned to implement this approach in the ongoing cohorts of the CogFrail (34) and INSPIRE Projects (35, 36, 37, 38, 39).

## Conclusion

At a biological level, age-related frailty and disease-related frailty may represent different degrees of frailty severity, with the latter cumulating the deleterious biological mechanisms leading to aging and disease development/progression. Further research is needed to identify biomarkers potentially able to distinguish these classifications of frailty at a biological level.

## MAPT/DSA Group

MAPT Study Group: Principal investigator: Bruno Vellas (Toulouse); Coordination: Sophie Guyonnet; Project leader: Isabelle Carrié; CRA: Lauréane Brigitte; Investigators: Catherine Faisant, Françoise Lala, Julien Delrieu, Hélène Villars; Psychologists: Emeline Combrouze, Carole Badufle, Audrey Zueras; Methodology, statistical analysis and data management: Sandrine Andrieu, Christelle Cantet, Christophe Morin; Multidomain group: Gabor Abellan Van Kan, Charlotte Dupuy, Yves Rolland (physical and nutritional components), Céline Caillaud, Pierre-Jean Ousset (cognitive component), Françoise Lala (preventive consultation) (Toulouse). The cognitive component was designed in collaboration with Sherry Willis from the University of Seattle, and Sylvie Belleville, Brigitte Gilbert and Francine Fontaine from the University of Montreal. Co-Investigators in associated centres: Jean-François Dartigues, Isabelle Marcet, Fleur Delva, Alexandra Foubert, Sandrine Cerda (Bordeaux); Marie-Noëlle-Cuffi, Corinne Costes (Castres); Olivier Rouaud, Patrick Manckoundia, Valérie Quipourt, Sophie Marilier, Evelyne Franon (Dijon); Lawrence Bories, Marie-Laure Pader, Marie-France Basset, Bruno Lapoujade, Valérie Faure, Michael Li Yung Tong, Christine Malick-Loiseau, Evelyne Cazaban-Campistron (Foix); Françoise Desclaux, Colette Blatge (Lavaur); Thierry Dantoine, Cécile Laubarie-Mouret, Isabelle Saulnier, Jean-Pierre Clément, Marie-Agnès Picat, Laurence Bernard-Bourzeix, Stéphanie Willebois, Iléana Désormais, Noëlle Cardinaud (Limoges); Marc Bonnefoy, Pierre Livet, Pascale Rebaudet, Claire Gédéon, Catherine Burdet, Flavien Terracol (Lyon), Alain Pesce, Stéphanie Roth, Sylvie Chaillou, Sandrine Louchart (Monaco); Kristelle Sudres, Nicolas Lebrun, Nadège Barro-Belaygues (Montauban); Jacques Touchon, Karim Bennys, Audrey Gabelle, Aurélia Romano, Lynda Touati, Cécilia Marelli, Cécile Pays (Montpellier); Philippe Robert, Franck Le Duff, Claire Gervais, Sébastien Gonfrier (Nice); Yannick Gasnier and Serge Bordes, Danièle Begorre, Christian Carpuat, Khaled Khales, Jean-François Lefebvre, Samira Misbah El Idrissi, Pierre Skolil, Jean-Pierre Salles (Tarbes). MRI group: Carole Dufouil (Bordeaux), Stéphane Lehéricy, Marie Chupin, Jean-François Mangin, Ali Bouhayia (Paris); Michèle Allard (Bordeaux); Frédéric Ricolfi (Dijon); Dominique Dubois (Foix); Marie Paule Bonceour Martel (Limoges); François Cotton (Lyon); Alain Bonafé (Montpellier); Stéphane Chanalet (Nice); Françoise Hugon (Tarbes); Fabrice Bonneville, Christophe Cognard, François Chollet (Toulouse). PET scans group: Pierre Payoux, Thierry Voisin, Julien Delrieu, Sophie Peiffer, Anne Hitzel, (Toulouse); Michèle Allard (Bordeaux); Michel Zanca (Montpellier); Jacques Monteil (Limoges); Jacques Darcourt (Nice). Medico-economics group: Laurent Molinier, Hélène Derumeaux, Nadège Costa (Toulouse). Biological sample collection: Bertrand Perret, Claire Vinel, Sylvie Caspar-Bauguil (Toulouse). Safety management: Pascale Olivier-Abbal. DSA Group: Sandrine Andrieu, Christelle Cantet, Nicola Coley.

## Electronic Supplementary Material

Blood markers assessment

Extra table: Time-point of measurement

## References

[1] Vetrano DL, Palmer K, Marengoni A, Marzetti E, Lattanzio F, Roller-Wirnsberger R, Lopez Samaniego L, Rodríguez-Mañas L, Bernabei R, Onder G (2019). Joint Action ADVANTAGE WP4 Group. Frailty and Multimorbidity: A Systematic Review and Meta-analysis. J Gerontol A Biol Sci Med Sci.

[2] Pandey A, Gilbert O, Kitzman DW. Physical frailty in older patients with acute heart failure: From risk marker to modifiable treatment target. J Am Geriatr Soc. 2021 Jun 19. doi: 10.1111/jgs.17306. Epub ahead of print. PMID: 34146340. PMC8440358 34146340

[3] Gill TM, Gahbauer EA, Han L, Allore HG (2011). The relationship between intervening hospitalizations and transitions between frailty states. J Gerontol A Biol Sci Med Sci.

[4] Landré B, Aegerter P, Zins M, Goldberg M, Ankri J, Herr M (2019). Association between Hospitalization and Change of Frailty Status in the GAZEL Cohort. J Nutr Health Aging.

[5] Takeda C, Angioni D, Setphan E, Macaron T, De Souto Barreto P, Sourdet S, Sierra F, Vellas B (2020). Age-Related Frailty: A Clinical Model for Geroscience?. J Nutr Health Aging.

[6] Angioni D, Macaron T, Takeda C, Sourdet S, Cesari M, Virecoulon Giudici K, Raffin J, Lu WH, Delrieu J, Touchon J, Rolland Y, de Souto Barreto P, Vellas B (2020). Can We Distinguish Age-Related Frailty from Frailty Related to Diseases? Data from the MAPT Study. J Nutr Health Aging.

[7] Rodriguez-Mañas L, Araujo de Carvalho I, Bhasin S, Bischoff-Ferrari HA, Cesari M, Evans W, Hare JM, Pahor M, Parini A, Rolland Y, Fielding RA, Walston J, Vellas B (2020). ICFSR Task Force Perspective on Biomarkers for Sarcopenia and Frailty. J Frailty Aging.

[8] Pérez-Ros P, Vila-Candel R, López-Hernández L, Martínez-Arnau FM (2020). Nutritional Status and Risk Factors for Frailty in Community-Dwelling Older People: A Cross-Sectional Study. Nutrients.

[9] Lu WH, Barreto PS, Rolland Y, Bouyahia A, Fischer C, Mangin JF, Giudici KV, Vellas B; MAPT/DSA Group. Biological and Neuroimaging Markers as Predictors of 5-year Incident Frailty in Older Adults: a Secondary Analysis of the MAPT study. J Gerontol A Biol Sci Med Sci. 2020 Nov 27:glaa296. doi: 10.1093/gerona/glaa296. Epub ahead of print. PMID: 33246338. 33246338

[10] Lu WH, de Souto Barreto P, Rolland Y, Rodríguez-Mañas L, Bouyahia A, Fischer C, Mangin JF, Giudici KV, Vellas B, MAPT/DSA Group (2020). Cross-sectional and prospective associations between cerebral cortical thickness and frailty in older adults. Exp Gerontol.

[11] Ferrucci L, Fabbri E (2018). Inflammageing: chronic inflammation in ageing, cardiovascular disease, and frailty. Nat Rev Cardiol.

[12] O’Keeffe M, Kelly M, O’Herlihy E, O’Toole PW, Kearney PM, Timmons S, O’Shea E, Stanton C, Hickson M, Rolland Y, Sulmont Rossé C, Issanchou S, Maitre I, Stelmach-Mardas M, Nagel G, Flechtner-Mors M, Wolters M, Hebestreit A, De Groot LCPGM, van de Rest O, Teh R, Peyron MA, Dardevet D, Papet I, Schindler K, Streicher M, Torbahn G, Kiesswetter E, Visser M, Volkert D, O’Connor EM, MaNuEL consortium (2019). Potentially modifiable determinants of malnutrition in older adults: A systematic review. Clin Nutr.

[13] Vassilaki M, Aakre JA, Mielke MM, Geda YE, Kremers WK, Alhurani RE, Machulda MM, Knopman DS, Petersen RC, Lowe VJ, Jack CR, Roberts RO (2016). Multimorbidity and neuroimaging biomarkers among cognitively normal persons. Neurology.

[14] Vellas B, Carrie I, Gillette-Guyonnet S (2014). Mapt Study: A Multidomain Approach for Preventing Alzheimer’s Disease: Design and Baseline Data. J Prev Alzheimers Dis.

[15] Carrie I, van Kan GA, Gillette-Guyonnet S (2012). Recruitment strategies for preventive trials. The MAPT study (MultiDomain Alzheimer Preventive Trial). J Nutr Health Aging.

[16] Andrieu S, Guyonnet S, Coley N (2017). Effect of long-term omega 3 polyunsaturated fatty acid supplementation with or without multidomain intervention on cognitive function in elderly adults with memory complaints (MAPT):a randomised, placebo-controlled trial. The Lancet Neurology.

[17] Fried LP, Tangen CM, Walston J, Newman AB, Hirsch C, Gottdiener J, Seeman T, Tracy R, Kop WJ, Burke G, McBurnie MA, Cardiovascular Health Study Collaborative Research Group (2001). Frailty in older adults: evidence for a phenotype. J Gerontol A Biol Sci Med Sci.

[18] Harris WS, Von Schacky C (2004). The Omega-3 Index: A new risk factor for death from coronary heart disease?. Prev Med (Baltim).

[19] Holick MF, Binkley NC, Bischoff-Ferrari HA (2011). Evaluation, treatment, and prevention of vitamin D deficiency: An endocrine society clinical practice guideline. J Clin Endocrinol Metab.

[20] Guo H, Chi J, Xing Y, Wang P (2009). Influence of folic acid on plasma homocysteine levels & arterial endothelial function in patients with unstable angina. Indian J Med Res.

[21] Giudici KV, de Souto Barreto P, Guerville F (2019). Associations of C-reactive protein and homocysteine concentrations with the impairment of intrinsic capacity domains over a 5-year follow-up among community-dwelling older adults at risk of cognitive decline (MAPT Study). Exp Gerontol.

[22] Operto G, Chupin M, Batrancourt B, Habert MO, Colliot O, Benali H (2016). CATI: A Large Distributed Infrastructure for the Neuroimaging of Cohorts. Neuroinformatics.

[23] Fleisher AS, Chen K, Liu X (2011). Using positron emission tomography and florbetapir F 18 to image cortical amyloid in patients with mild cognitive impairment or dementia due to Alzheimer disease. Arch Neurol.

[24] Assadi A, Zahabi A, Hart RA (2020). GDF15, an update of the physiological and pathological roles it plays: a review. Pflugers Arch.

[25] Emmerson PJ, Duffin KL, Chintharlapalli S, Wu X (2018). GDF15 and growth control. Frontiers in physiology.

[26] Coll AP, Chen M, Taskar P, Rimmington D, Patel S, Tadross JA, Cimino I, Yang M, Welsh P, Virtue S, Goldspink DA, Miedzybrodzka EL, Konopka AR, Esponda RR, Huang JTJ, Tung YCL, Rodriguez-Cuenca S, Tomaz RA, Harding HP, Melvin A, Yeo GSH, Preiss D, Vidal-Puig A, Vallier L, Nair KS, Wareham NJ, Ron D, Gribble FM, Reimann F, Sattar N, Savage DB, Allan BB, O’Rahilly S (2019). GDF15 mediates the effects of metformin on body weight and energy balance. Nature.

[27] Hanlon P, Nicholl BI, Jani BD, Lee D, McQueenie R, Mair FS (2018). Frailty and pre-frailty in middle-aged and older adults and its association with multimorbidity and mortality: a prospective analysis of 493 737 UK Biobank participants. Lancet Public Health.

[28] Guerville F, De Souto Barreto P, Ader I, Andrieu S, Casteilla L, Dray C, Fazilleau N, Guyonnet S, Langin D, Liblau R, Parini A, Valet P, Vergnolle N, Rolland Y, Vellas B (2020). Revisiting the Hallmarks of Aging to Identify Markers of Biological Age. J Prev Alzheimers Dis.

[29] Watanabe Y, Tatsuno I (2020). Prevention of Cardiovascular Events with Omega-3 Polyunsaturated Fatty Acids and the Mechanism Involved. J Atheroscler Thromb.

[30] Sharma T, Mandal CC (2020). Omega-3 fatty acids in pathological calcification and bone health. J Food Biochem.

[31] Avallone R, Vitale G, Bertolotti M (2019). Omega-3 Fatty Acids and Neurodegenerative Diseases: New Evidence in Clinical Trials. Int J Mol Sci.

[32] Rea IM, Gibson DS, McGilligan V, McNerlan SE, Alexander HD, Ross OA (2018). Age and Age-Related Diseases: Role of Inflammation Triggers and Cytokines. Front Immunol.

[33] Rodriguez-Mañas L, Araujo de Carvalho I, Bhasin S, Bischoff-Ferrari HA, Cesari M, Evans W, Hare JM, Pahor M, Parini A, Rolland Y, Fielding RA, Walston J, Vellas B (2020). ICFSR Task Force Perspective on Biomarkers for Sarcopenia and Frailty. J Frailty Aging.

[34] Sourdet S, Soriano G, Delrieu J, Steinmeyer Z, Guyonnet S, Saint-Aubert L, Payoux P, Ousset PJ, Ghisolfi A, Chicoulaa B, Dardenne S, Gemar T, Baziard M, Guerville F, Andrieu S, Vellas B (2021). Cognitive Function and Amyloid Marker in Frail Older Adults: The COGFRAIL Cohort Study. J Frailty Aging.

[35] Guyonnet S, Rolland Y, Takeda C, Ousset P-J, Ader I, Davezac N, et al. The INSPIRE Bio-Resource Research Platform for Healthy Aging and Geroscience: Focus on the Human Translational Research Cohort (The INSPIRE-T Cohort). J Frailty Aging [Internet]. 10 juill 2020 10.14283/jfa.2020.38. PMC7352084 33575699

[36] Takeda C, Guyonnet S, Sumi Y, Vellas B, Araujo de Carvalho I (2020). Integrated Care for Older People and the Implementation in the INSPIRE Care Cohort. J Prev Alzheimers Dis.

[37] de Souto Barreto P, Guyonnet S, Ader I, et al. The Inspire Research Initiative: A Program for Geroscience and Healthy Aging Research going from Animal Models to Humans and the Healthcare System. J Frailty Aging: 10.14283/jfa.2020.18. 33575696

[38] N. Tavassoli, A. Piau, C. Berbon, J. De Kerimel, C. Lafont, P. De Souto Barreto, et al. Framework Implementation of the INSPIRE ICOPE-CARE program in collaboration with the World Health Organization (WHO) in the Occitania region. J Frailty Aging: 10.14283/jfa.2020.26. 33575698

[39] Santin Y, Lopez S, Ader I, Andrieu S, Blanchard N, Carriere A et Al. Towards a large scale assessment of the relationship between biological and chronological aging: The Inspire mouse cohort. J Frailty Aging 2020 Published online August 7, 2020, 10.14283/jfa.2020.43. 33575700

