# Supplementary material for: Biomarkers of Age-Related Frailty and Frailty Related to Diseases: An Exploratory, Cross-Sectional Analysis from the MAPT Study
Source: J Nutr Health Aging. 2022 May 4;26(6):545–51. doi: 10.1007/s12603-022-1793-9 (PMC12878587; doi:10.1007/s12603-022-1793-9)
Supplement: Supplementary file 1 — Blood markers assessment [file mmc1.docx]

**SUPPLEMENTARY MATERIAL**

**Blood markers assessment**

**Fatty acids.** Lipids were extracted from red blood cells after acidification by using a mixture of hexane and isopropanol; Margaric acid was added as an internal standard. Total lipid extracts were saponified and methylated. Fatty acid methyl esters were extracted with pentane and analyzed by gas chromatography using an Agilent Technologies 6890N gas chromatograph with a split injector, a bonded silica capillary column, and a flame ionization detector. Identification of fatty acid methyl esters was based on retention times obtained for FAME prepared from fatty acid standards. The area under the curve (AUC) was measured using ChemStation software (Agilent) and expressed as a percent of total fatty acids.

**ApoE4:** PAX gene tubes were utilized for blood collection and the QIAamp 96 DNA QIAcube HT Kit (Qiagen, France) was used for [genomic DNA](https://www.sciencedirect.com/topics/medicine-and-dentistry/genomic-dna) extraction. In order to determine the ApoE genotype [pyrosequencing](https://www.sciencedirect.com/topics/medicine-and-dentistry/pyrosequencing) analysis was performed through the use of a Pyromark PCR kit (Qiagen, France) according the manufacturer's instructions.

**Aβ assessment.** Targeted Aβ isoforms (Aβ38, Aβ40, and Aβ42) were simultaneously immunoprecipitated from 0.45 mL of plasma via a monoclonal anti-Aβ mid-domain antibody (HJ5.1, antiAβ13-28) conjugated to M-270 Epoxy Dynabeads (Invitrogen). Previous to immunoprecipitation, samples were spiked with a known quantity of 12C15N-Aβ38, 12C15N-Aβ40 and 12C15N-Aβ42 for use as analytical internal standards. Proteins were digested into peptides using LysN endoprotease. Liquid chromatography-mass spectrometry was performed. Plasma analyses were realized as targeted parallel reaction monitoring on an Orbitrap Fusion Lumos Tribrid mass spectrometer (Thermo Fisher) interfaced with an M-class nanoAcquity chromatography system (Waters). The derived integrated peak areas were thus analyzed by using the Skyline software package. Aβ42 and Aβ40 levels were measured by integrated peak area ratios to known concentrations of the internal standards. The value of Aβ42/40 ratio (dividing plasma Aβ42 by Aβ40) was then calculated and their normalized values were used.

**Neurofilaments.** Samples were diluted 4-fold in diluent buffer and tested in duplicate. Plasma neurofilaments were measured using the R-PLEX human neurofilament L antibody set (Meso Scale Discovery, F217X). Samples were diluted two-fold in diluent buffer and tested in duplicate.

**Progranulin assessment.** Samples were diluted 2-fold in a diluent buffer and assayed in duplicate following to the manufacturer’s instructions. Plasma PGRN levels were measured by a commercially available ELISA kit (R&D Systems, DPGRN0). Samples were diluted 4-fold in diluent buffer and assayed in duplicate according to the manufacturer’s instructions

**Grow differentiation factor 15, Tumor necrosis factor receptor 1, Interleukin 6, Monocyte chemoattractant protein 1.** Proteins levels were measured by using a single disposable microfluidic SimplePlexTM cartridge. The plasma samples were thawed on ice and diluted 1-4 in sample diluent (SD 13) then loaded into cartridges with relevant high and low control concentrates. Within the cartridge each plasma sample was divided into 4 unique microfluidic parallel channels (that are specific for each of the four proteins analyzed). Every protein channel contains 3 analyte-specific glass nanoreactors that allows for each plasma sample to be run in triplicates for each of the four protein samples. Cartridges include a built-in lot-specific standard curve for each protein. All steps previously described were performed automatically by the instrument without user activity. The obtained protein concentrations were displayed in pg/mL and calculated by the internal instrument software.
