# Supplementary material for: Biomarkers of Age-Related Frailty and Frailty Related to Diseases: An Exploratory, Cross-Sectional Analysis from the MAPT Study
Source: J Nutr Health Aging. 2022 May 4;26(6):545–51. doi: 10.1007/s12603-022-1793-9 (PMC12878587; doi:10.1007/s12603-022-1793-9)
Supplement: Supplementary file 2 — Extra table: Time-point of measurement [file mmc2.docx]

**Extra table: Time-point of measurement**

|  | **time-point** |
| --- | --- |
| ***Plasma markers*** |  |
| 25-hydroxyvitamin D (ng/mL) | Baseline |
| Homocysteine (μmol/L) | Baseline |
| Erythrocyte membrane fatty acid | Baseline |
| CRP (mg/L) | Baseline, 6 month and 1 year |
| ApoE ε4 carrier | Baseline |
| Aβ_42/40_ ratio | 12 months |
| Neurofilament light chain (pg/mL) | 12 months |
| Progranulin (ng/mL) | 12 months |
| GDF15 (pg/mL) | 12 months |
| TNFR1 (pg/mL) | 12 months |
| IL6 (pg/mL) | 12 months |
| MCP1 (pg/mL) | 12 months |
| ***Imaging markers*** |  |
| Cortical SUVR | Between baseline and 3 years |
| Gray matter volume (cm^3^) | Between baseline and 3 years |
| Hippocampal volume (cm^3^) | Between baseline and 3 years |
| WMH volume (cm^3^) | Between baseline and 3 years |
| Cortical thickness, whole brain (mm) | Between baseline and 3 years |

Abbreviation: Aβ, amyloid-beta; GDF15, growth differentiation factor 15; IL6, interleukin 6; MCP1, monocyte chemoattractant protein 1; SUVR, standard uptake value ratio; TNFR1, tumor necrosis factor receptor 1; WMH, white matter hyperintensities.
